# Soil hypoxia induced by an organic-material mulching technique stimulates the bamboo rhizome up-floating of *Phyllostachys praecox*

**DOI:** 10.1038/s41598-017-14798-8

**Published:** 2017-10-30

**Authors:** Mengjie Xu, Shunyao Zhuang, Renyi Gui

**Affiliations:** 1 College of Public Administration, Nanjing Agriculture University, Nanjing, 210095 P. R. China; 2 State Key Lab of Soil and Sustainable Agriculture, Institute of Soil Science, Chinese Academy of Sciences, Nanjing, 210008 P. R. China; 3 State Key Lab of Subtropical Forest Silviculture, Zhejiang Agriculture & Forestry University, Lin’an, 311300 P. R. China

## Abstract

*Phyllostachys praecox* bamboo stands significantly recede after 3 or 4 years using an organic-material mulching technique consecutively. We hypothesized that the bamboo recession is caused by the up-floating of underground rhizome stimulated by soil hypoxia through the mulching technique. This study aimed to validate this hypothesis by field investigation. Bamboo underground rhizome distribution in the soil profile of *P. praecox* subjected to various mulching times was investigated. Results showed that bamboo rhizome weights and lengths increased with increased mulching time. However, after 4 years of mulching, the number of fresh rhizomes decreased significantly, and more than 50% of rhizomes floated upward to the shallow soil layer (0–10 cm). Moreover, the 0–10 cm soil layer suffered severe acidification that severely impeded bamboo-rhizome growth. The soil hypoxia induced by the mulching technique must be responsible for the bamboo rhizome up-floating. We confirmed that bamboo rhizome up-floating was the critical factor that caused the bamboo growth to recede under the mulching technique. Therefore, managing this bamboo rhizome up-floating is the key to sustainable bamboo production. The effect of soil hypoxia in the absence of flooding or waterlogging on plant root growth also warrants further and extensive study.

## Introduction

With modern agricultural progress, intensive crop management, such as fertilization, deinsectization, irrigation, and continuous cropping, has greatly influenced crop growth, yield, and quality. However, many concerns have arisen with intensive management and impeded sustainable production. *Phyllostachys praecox* is a kind of bamboo (Bambusoideae) cultivated to produce edible bamboo shoots. It is distributed extensively across southeast China, such as in Zhejiang, Anhui, Fujiang, and Jiangxi provinces. Given its high quality and economic profit, the bamboo is intensively cultivated by organic material mulching^1^. This technique was first developed in the 1980s in Lin’an City, Zhejiang Province, China. Usually, farmers allow rice straw and animal manure to ferment on soil surfaces on November or December to increase the soil temperature during the winter. Then, a 30 cm layer containing rice bran is mulched on the surface to preserve the fermentation heat. The rice bran layer is then removed on March or April of the next year. When using the technique, the bamboo shoot can be harvested before the Chinese Spring Festival and sold for a good market price. Bamboo farmers gain high economic profits through bamboo cultivation. For example, the total bamboo shoot income in Lin’an City reached RMB 919 million (equal to US $150 million) in 2014; the output-on-area basis was close to $30,000 ha^−1^ ^2^. Therefore, organic material mulching has been extensively adopted in *P. praecox* production, and bamboo production became an important rural supporting industry. However, after several years of continuous mulching, the bamboo inevitably showed significant recession. The bamboo recession mainly presented as low shoot yield that decreased from 37,500 kg ha^−1^ to less than 15,000 kg ha^−1^. As surveyed, only 1/4 of the bamboo area can be mulched every year in Lin’an City^3^. Such common phenomenon indicated that the bamboo recession is critical to the sustainable production by organic material mulching. When the technique was not used, no bamboo recession was observed. However, the technique enabled the bamboo farmers to gain economic profit. Therefore, this bamboo recession problem under the mulching technique must be resolved.

Given the above-mentioned consequence, bamboo recession has been paid with increased attention. Many researchers conducted various studies to explore the mechanism of the bamboo recession caused by the mulching technique. According to Zhou *et al*.^4^, the bamboo abundance receded because the mulching materials are not removed in time, and the mother bamboo is left needlessly. Meanwhile, Jiang *et al*.^5^ suggested that the high phosphorus accumulation in soil (soil available phosphorus reached 130 mg kg^−1^) resulted in bamboo flowering and led to the bamboo recession. By contrast, Yu *et al*.^6^ hypothesized that the imbalance in nutrient supply and demand, as well as physiological disorder, is responsible for the bamboo recession. Zheng^7^ pointed out that the amount of phenolic acid that decomposes from the mulching material is higher than that from the control by 39.0% and might influence bamboo growth. Gui *et al*.^3^ found that soil acidification was severe and soil active Al content was very high (increased from 3.8 to 197.6 mg kg^−1^), which can be harmful to bamboo growth. Chen *et al*.^2^ observed that the bamboo root is modestly capable of assimilating nutrients under long-term mulching application, thus weakening the bamboo stand renewal. All these factors may cause bamboo recession, but none of them can be responsible for the underlying mechanism. To date, not one effective measure has been used to solve the challenges in practice, and the bamboo stand tends to recede inevitably after using the technique continuously for three or four times. Obviously, the critical mechanism has not been discovered.

Given the field investigation, we hypothesized that the bamboo recession was closely related to the underground rhizome and the soil associated with the mulching technique. The mulching technique induces hypoxia in the bamboo soil due to organic material fermentation, which then stimulates the bamboo rhizome to float upward. Therefore, in this study, we selected a series of bamboo stands would undergo various mulching times to investigate their underground bamboo rhizomes and soils. We aimed to thoroughly understand the bamboo recession induced by the mulching technique and provide a basis for countermeasures. Moreover, such a study would be interesting worldwide for a new understanding of soil hypoxia in absence of flooding or waterlogging.

## Results

### Soil basic properties associated with the mulching technique

The soil pH in the 0–10 cm layer ranged from 4.02 to 4.30 without significant difference among various treatments (Table 1). This result was similar to that of the 10–20 cm layer. However, the soil pH was much higher at the 20–40 cm layer than those at 0–20 cm. Thus, soil pH increased with soil depth, and the surface soil of 0–20 cm was acidified severely (pH < 4.5). Soil organic matter (SOM) content ranged from 31.13 to 64.17 mg kg^−1^ in the surface layer of 0–10 cm. The SOM almost increased with mulching time and increased to the highest content in T3 at 64.17 g kg^−1^. In the soil profiles, soil organic matter decreased with soil depth in all the treatments. CEC displayed the same pattern as SOM in all the treatments and profiles, but the largest CEC was noted at T4 as 26.58 cmol_(+)_ kg^−1^.

**Table 1:** Variation in the basic properties of the soil profile with mulching time*.

Treatment	Soil layer (cm)	pH	Organic matte content (g·kg^−1^)	Cation exchange capacity (cmol_(+)·_kg^−1^)
T0	0–10	4.19 ± 0.11 b	31.13 ± 4.17 cd	18.35 ± 1.13 c
	10–20	4.40 ± 0.19 b	28.93 ± 2.35 de	17.03 ± 0.49 cd
	20–40	5.79 ± 0.27 a	25.00 ± 1.47 de	15.53 ± 0.38 d
T1	0–10	4.22 ± 0.12 b	35.13 ± 4.17 c	19.31 ± 1.32 c
	10–20	4.35 ± 0.18 b	31.21 ± 2.35 de	17.34 ± 0.65 cd
	20–40	5.64 ± 0.25 a	24.23 ± 2.12 de	15.51 ± 0.41 d
T2	0–10	4.11 ± 0.13 b	46.11 ± 3.17 b	20.35 ± 0.94 bc
	10–20	4.20 ± 0.17 b	33.24 ± 2.26 cd	17.83 ± 0.54 cd
	20–40	5.61 ± 0.18 a	25.08 ± 2.32 de	15.56 ± 0.68 d
T3	0–10	4.30 ± 0.14 b	64.17 ± 9.57 a	22.86 ± 2.76 b
	10–20	4.21 ± 0.23 b	37.53 ± 2.03 bc	18.91 ± 1.23 c
	20–40	5.64 ± 0.11 a	26.07 ± 4.86 de	16.89 ± 1.01 cd
T4	0–10	4.02 ± 0.19 b	62.73 ± 0.60 a	26.58 ± 0.73 a
	10–20	4.11 ± 0.11 b	41.10 ± 0.82 b	21.48 ± 0.46 b
	20–40	5.54 ± 0.77 a	23.30 ± 1.91 e	15.55 ± 0.87 d

*The data were presented as mean ± SD. The various letters represent significant difference (P = 0.05) under the LSD method. The same caption applies below.

### Bamboo rhizoNme number, weight, and length

**Figure 1:**
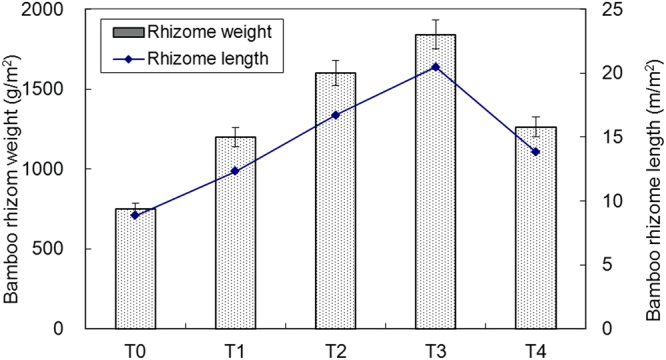
Bamboo rhizome weight and length varying with mulching time.

The bamboo stand structure was influenced greatly by the mulching technique. The bamboo density increased from T0 13,200 culm ha^−1^ to T3 15,000 culm ha^−1^ over the 3 years of mulching but decreased to 9,000 culm ha^−1^ at the fourth year (T4) (Table 2). By contrast, the bamboo diameter at breast height (DBH) increased with mulching time from 3.66 cm to 4.83 cm. Before using the mulching technique (T0), the bamboo underground rhizome weight was 750 g m^−2^, and the rhizome length was 8.84 m m^−2^ (Fig. 1). With increased mulching time, the bamboo rhizome weight and length rose to 1841 g m^−2^ and 20.5 m m^−2^, respectively, on the third year. However, the bamboo rhizome weight and length decreased significantly on the fourth year (Fig. 1). These results indicated that the mulching technique can be used for only three times at most; otherwise, the bamboo stand would be damaged irreversibly.

**Table 2:** Site description of the bamboo stands with various mulching times.

	T0	T1	T2	T3	T4
Stand density (culm ha^−1^)	13,200 ± 145 c	14,500 ± 150 ab	14,800 ± 157 a	15,000 ± 145 a	9,000 ± 101 d
Bamboo DBH (cm)	3.66 ± 0.11 d	3.87 ± 0.12 c	4.52 ± 0.13 b	4.77 ± 0.14 a	4.83 ± 0.13 a

### Composition of fresh and old bamboo rhizome

**Figure 2:**
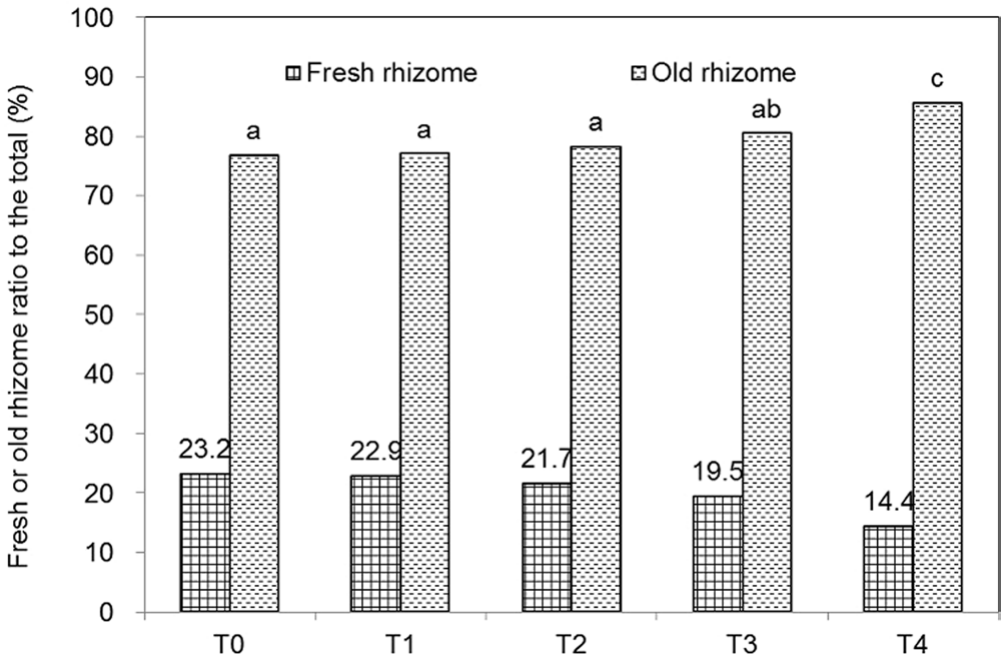
Fresh bamboo rhizome vs. old rhizome composition.

The bamboo underground fresh rhizome in the nonmulched plot was 23.2% of the total rhizome in weight, but decreased significantly to 14.4% at the fourth year with mulching (Fig. 2). By contrast, the old rhizome percentage increased from 76.8% to 85.6%. These results suggested that the bamboo rhizome progressively weakened with mulching time.

### Bamboo rhizome distribution along soil depth

**Figure 3:**
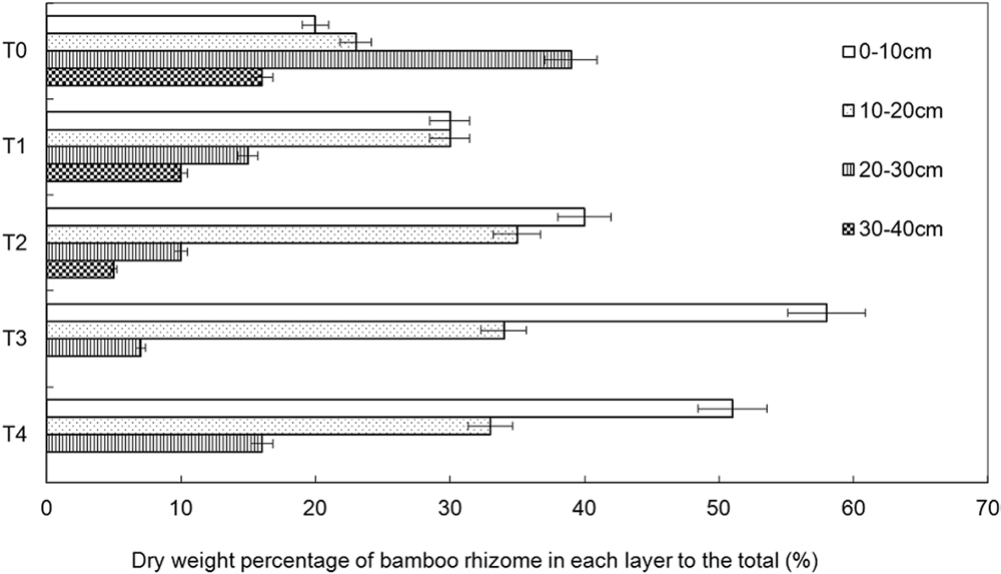
Bamboo rhizome distribution in the soil profile.

The distribution of the bamboo underground rhizome was significantly affected by the mulching technique. The rhizome content in the 0–10 cm layer increased from 20% to 58% with time (Fig. 3). However, the rhizome content in part of the > 30 cm layer decreased dramatically from 16% to 0% after 3 years. Obviously, the distribution of bamboo rhizome in the soil profile floated up from deep to shallow soil layers. Specifically, the organic-matter mulching technique stimulated the bamboo-rhizome growth to float upward. Because bamboo roots develop from the rhizome, the up-floated rhizome brings the bamboo root to a much shallow soil layer; this occurrence substantially harms bamboo growth.

## Discussion

Through the organic-matter mulching technique, bamboo shoot production and bamboo farmer income were improved considerably. Without the technique, no profit can be obtained. Accordingly, the technique has become an essential method for bamboo production. However, some negative aspects induced by the technique are obvious and inevitable. As reported^2,8,9^, soil acidification aggravates when the technique is applied to *P. praecox* because of increased chemical fertilizer input. Our results also showed the same phenomenon (Table 1). The severe soil acidification is a potential crucial factor influencing the bamboo recession. However, soil acidification mainly occurs in the surface layer (0–10 cm), whereas the bamboo root distributes in the deep soil layers (20–40 cm). Therefore, soil acidification unlikely causes the bamboo recession.

Bamboo rhizome is a complex underground system that germinates bamboo shoots and roots. That is, the development of bamboo shoots from rhizome buds is very special and particularly significant to bamboo productivity^10^. Bamboo shoot germination is determined by miRNA genetics but is also influenced by natural factors^11^. Usually, a rhizome bud can either develop into a bamboo shoot vertically in spring or into a rhizome shoot horizontally in the summer. The bamboo rhizome up-floating is likely related to the rhizome shoot growth rather than bamboo shoot growth. Hence, the temperature increase caused by the mulching technique is not due to the bamboo rhizome up-floating. Similarly, the normal bamboo cultivation without mulching would not result in bamboo rhizome up-floating in the high temperatures of summer.

Organic-material mulching may induce soil hypoxia during the warming period because of the decomposing organic material. However, such hypoxia has not been noted and related to bamboo growth. As reported^12,13^, each plant species manifests a varied tolerance degree and duration to low oxygen. After prolonged asphyxiation periods, visible symptoms, such as wilting, followed by defoliation of the aerial part, appear before the lethal phase. The resistance to root asphyxiation varies depending on the plant species (e.g., from a few days for lupine to a few weeks for wheat)^14^. Plants can recover their normal physiological activities if root aeration is restored before the lethal phase. As for *P. praecox*, the mulching period usually lasts for 3–4 months each year. Such a long hypoxic duration is lethal to most plant species. However, the hypoxia in the bamboo stand differs from the oxygen depletion from waterlogging or soil compaction, overirrigation, or poor drainage^15–17^. The response of *P. praecox* to such soil hypoxia also varies from other cases under waterlogging conditions. The mechanisms of the bamboo response to soil hypoxia are still unknown and merit further studies.

Our results showed that the organic material mulching technique improved the bamboo shoot production initially, but weakened the bamboo rhizome after 3–4 years. The up-floated and weakened bamboo rhizome resulted in the bamboo recession inevitably. Therefore, controlling bamboo rhizome up-floating with intensive management is the key to sustaining bamboo production.

## Methods

### Study site description

The study site was located at Taihuyuan Town, Lin’an City, Zhejiang Province, China (119°33′14″E, 30°16′53″N). This site belongs to the north subtropical monsoon region with a rainfall of 1420 mm, mean temperature of 15.2 °C, annual sunshine time of 1939 h, and 234 d of frost-free period per year. The landform of the agricultural field is a hilly landscape, with hills mostly lower than 150 m. Soil is mostly derived from quaternary sandstone parent material and classified as Ferralsols. *P. praecox* is cultivated for bamboo shoot production, which is a major economic source for local farmers. Lin’an City produces the largest *P. praecox* cultivation area in China. The bamboo area reached around 30,000 ha in 2014.

### Organic-material mulching technique

The organic material mulching technique used on *P. praecox* has been extensively adopted in bamboo production. In brief, we describe the procedures as follows. Five years after bamboo transplanting, the canopy of a *P. praecox* bamboo stand is closed, and the stands become ready for commercial shoot production. To harvest bamboo shoots early, farmers developed organic material mulching by using materials, such as straw, bran, and sometimes bamboo leaves^1^. Mulching usually starts from November or December and lasts until March of next year to increase the soil temperature by 4 °C–5 °C and conserve soil moisture in the winter season^18^. After March, the mulching material are removed as much as possible and piled beside the bamboo plot. As reported, the lost weight of mulching material was about 1/3 every time^3^. In the mulching process, rice straw is initially applied as mulch to 10–15 cm thickness on the soil surface. Then, rice bran is added on top to an additional 10–15 cm thickness. The total amount of rice straw and bran used at a time reaches a rate of 95 Mg ha^−1^ yr^−1^ (40 Mg rice straw ha^−1^ yr^−1^ and 55 Mg rice bran ha^−1^ yr^−1^). The mulching technique is used since the fifth year after bamboo transplanting. Under the technique, a high fertilization rate (1,125 kg ha^−1^ urea and 2,250 kg ha^−1^ complex fertilizer (N–P–K: 16–16–16) is also adopted for 1 year before mulching.

### Soil and bamboo rhizome sampling

In this study, a series of bamboo stands that would involve various mulching times was selected. Usually, the continuous mulching time is less than 4 years because the bamboo stand shows great recession after 4 years under the mulching technique. The bamboo mulching times were 0, 1, 2, 3, and 4 years, marked as T0, T1, T2, T3, and T4, respectively. Three plots of the same mulching time were sample for soil and bamboo rhizome on May 2015. Each plot measured more than 0.1 ha. In each plot, soil samples were collected in triplicate in three layers, i.e., 0–10, 10–20, and 20–40 cm. Five small subplots of 1 m × 1 m were excavated to collected bamboo rhizome in four layers (0–10, 10–20, 20–30, and 30–40 cm). The bamboo rhizome was then identified with old and fresh parts. The old bamboo rhizome has no bamboo shoot buds for germination and has a significant appearance in lignification. While the fresh part is active with bamboo shoot buds and formed in one or two years. Each bamboo rhizome part was sized and weighed. Furthermore, each bamboo plot was investigated culm by culm, and the bamboo number was recorded.

### Sample analysis

The soil sample pH was measured in distilled water (soil to solution = 1:2.5). Soil organic matter was measured by the wet digestion method. Meanwhile, the cation exchange capacity (CEC) of the soils was determined by the indophenol blue colorimetric method, where soils were exchanged by 1 M NH_4_–acetate solution with pH 7.0^19^. The bamboo rhizomes collected from the field were washed and oven dried at 65 °C. The dried bamboo rhizomes were weighed by electronic balance.

### Data statistics

The data were analyzed by ANOVA through SPSS. Fisher’s least significant difference (LSD) test of one-way ANOVA was performed to determine the significance of the differences recorded among various treatments and the horizons in the bamboo plots.

### Data availability

All relevant data are within the paper and its Supporting Information files.

## Electronic supplementary material

Supplementary information
