# Supplementary material for: Soil hypoxia induced by an organic-material mulching technique stimulates the bamboo rhizome up-floating of Phyllostachys praecox
Source: Sci Rep. 2017 Oct 30;7:14353. doi: 10.1038/s41598-017-14798-8 (PMC5662596; doi:10.1038/s41598-017-14798-8)
Supplement: Supplementary file 1 — Supplementary information [file 41598_2017_14798_MOESM1_ESM.pdf]

Supplementary Information

Title:

Soil hypoxia induced by an organic-material mulching technique stimulates the bamboo rhizome up-floating of *Phyllostachys praecox*

Authors:

Mengjie Xu[1], Shunyao Zhuang[2], Renyi Gui[3]

Affiliations:

1 College of Public Administration, Nanjing Agriculture University, Nanjing 210095, PR China

2 State Key Lab of Soil and Sustainable Agriculture, Institute of Soil Science, Chinese Academy of Sciences, Nanjing 210008, PR China

3 State Key Lab of Subtropical Forest Silviculture, Zhejiang Agriculture & Forestry University, Lin'an 311300, PR China

Corresponding author:

Dr. SY Zhuang

Email-address: syzhuang@issas.ac.cn

Use of the organic material mulching technique in site

The photos listed below are the bamboo stand and shoot pictures using the organic material mulching technique.

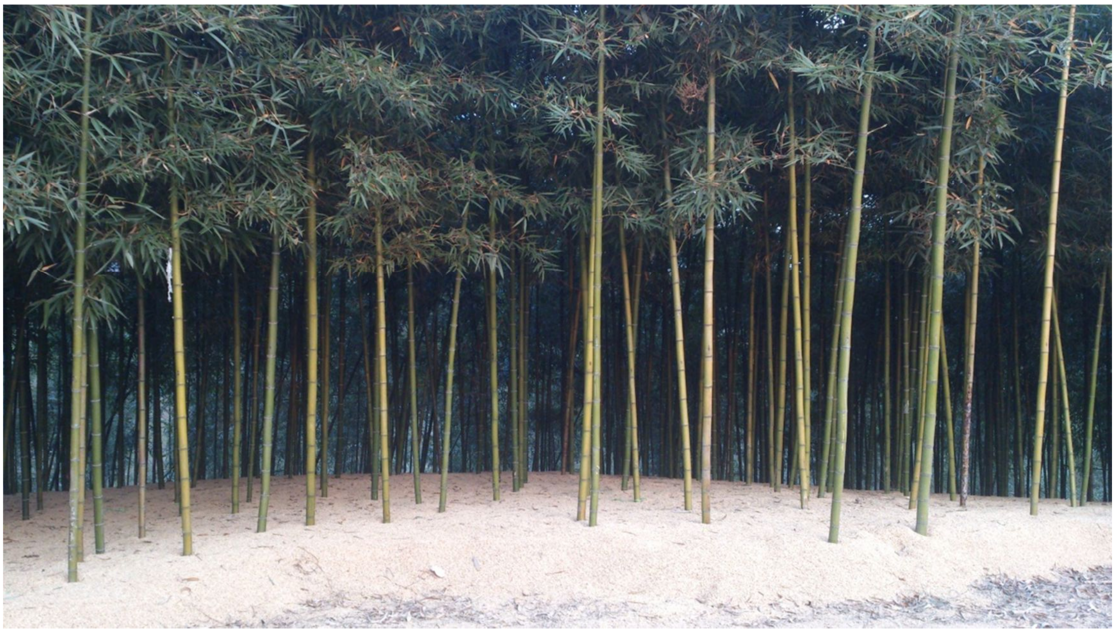

Fig. 1 *Phyllostachys praecox* propagated through the organic material mulching technique (Photo taken by Dr. Zhuang at Lin-an city, Zhejiang province of China)

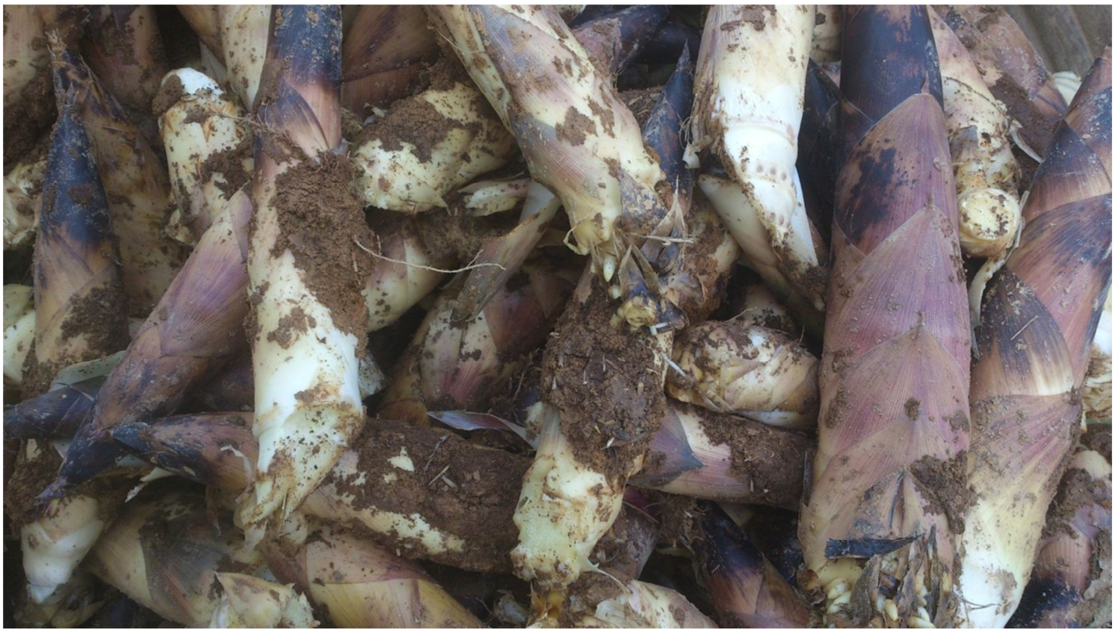

Fig. 2 Bamboo shoots collected in the field (Photo was taken by Dr. Zhuang at Lin-an city, Zhejiang province of China)

Evidences of soil hypoxia using the organic material mulching technique

Fig. 3 and Fig. 4 showed that soil hypoxia condition was significant when using the organic material mulching technique in the bamboo field. These data were obtained from the extra experiment in the same site.

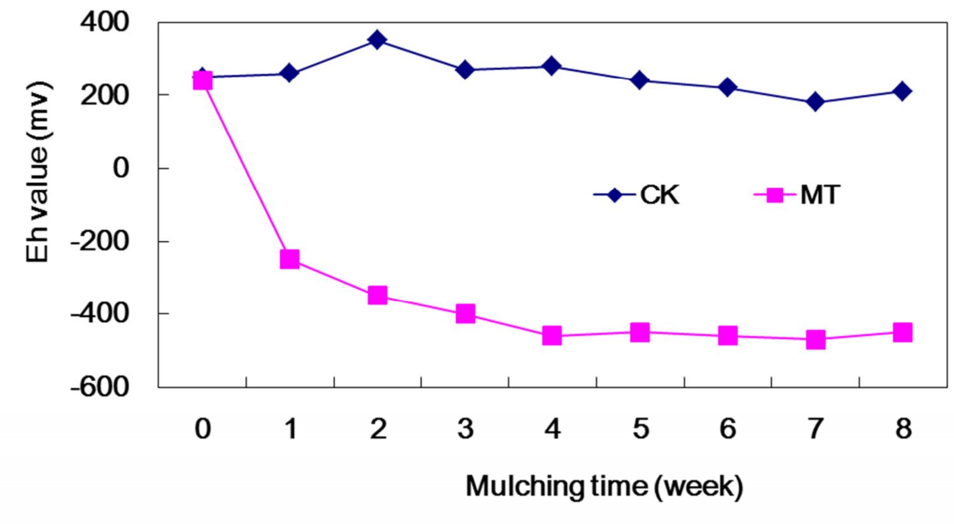

Fig. 3 Soil Eh value dynamics varied with the mulching time (unpublished data)

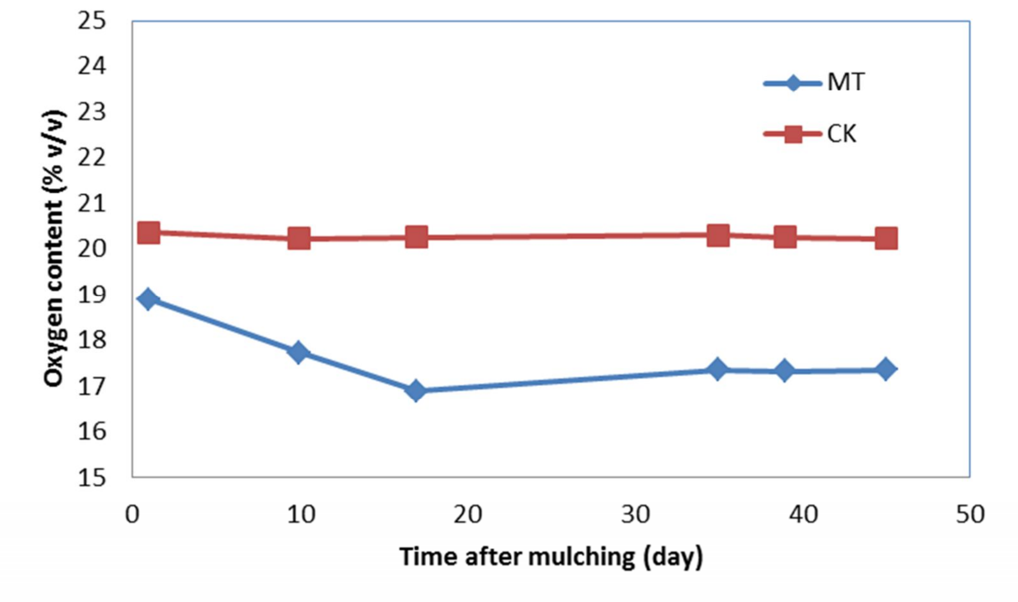

Fig. 4 Soil oxygen content during the mulching time (unpublished data)
